# A survey of open questions in adaptive therapy: Bridging mathematics and clinical translation

**DOI:** 10.7554/eLife.84263

**Published:** 2023-03-23

**Authors:** Jeffrey West, Fred Adler, Jill Gallaher, Maximilian Strobl, Renee Brady-Nicholls, Joel Brown, Mark Roberson-Tessi, Eunjung Kim, Robert Noble, Yannick Viossat, David Basanta, Alexander RA Anderson

**Affiliations:** 1 Department of Integrated Mathematical Oncology, H. Lee Moffitt Cancer Center & Research Institute Tampa United States; 2 Department of Mathematics, University of Utah Salt Lake City United States; 3 School of Biological Sciences, University of Utah Salt Lake City United States; 4 Natural Product Informatics Research Center, Korea Institute of Science and Technology Gangneung Republic of Korea; 5 Department of Mathematics, University of London London United Kingdom; 6 Ceremade, Université Paris-Dauphine, Université Paris Sciences et Lettres Paris France; Memorial Sloan Kettering Cancer Center United States; Memorial Sloan Kettering Cancer Center United States

**Keywords:** adaptive therapy, mathematical modeling, drug resistance, cancer evolution & evolution, predictive modeling

## Abstract

Adaptive therapy is a dynamic cancer treatment protocol that updates (or ‘adapts’) treatment decisions in anticipation of evolving tumor dynamics. This broad term encompasses many possible dynamic treatment protocols of patient-specific dose modulation or dose timing. Adaptive therapy maintains high levels of tumor burden to benefit from the competitive suppression of treatment-sensitive subpopulations on treatment-resistant subpopulations. This evolution-based approach to cancer treatment has been integrated into several ongoing or planned clinical trials, including treatment of metastatic castrate resistant prostate cancer, ovarian cancer, and BRAF-mutant melanoma. In the previous few decades, experimental and clinical investigation of adaptive therapy has progressed synergistically with mathematical and computational modeling. In this work, we discuss 11 open questions in cancer adaptive therapy mathematical modeling. The questions are split into three sections: (1) integrating the appropriate components into mathematical models (2) design and validation of dosing protocols, and (3) challenges and opportunities in clinical translation.

## Introduction

*Jeffrey West, Eunjung Kim, Rob Noble, Yannick Viossat, David Basanta, Alexander Anderson*: Treatment resistance in cancer therapy remains an overarching challenge across all types of cancer and all modes of treatment including targeted therapy, chemotherapy, and immunotherapy. Despite the ubiquity of the evolution of resistance, the ‘more is better’ paradigm still prevails as standard of care. Over the past decade, a small group of oncologists in collaboration with evolutionary biologists and experimental biologists have proposed an ‘adaptive therapy’ approach to cancer treatment (Gatenby et al., 2009b; Zhang et al., 2017; Zhang et al., 2022). Adaptive therapy maintains high levels of tumor burden in order to capitalize on competition between treatment-sensitive and treatment-resistant clones, and the potential cost of resistance.

**Figure 1.**
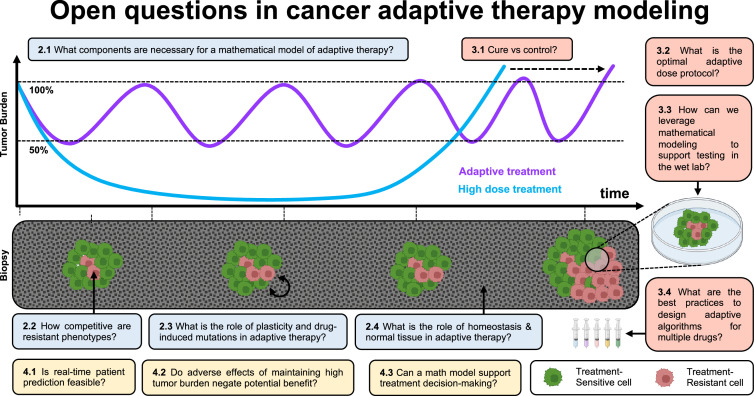
Open questions in adaptive cancer therapy modeling: schematic of tumor burden under maximum tolerable dose (blue) and adaptive dosing (purple), with corresponding biopsies. Adaptive therapy is designed to exploit competition between treatment-sensitive (green) and resistant (red) cells to prolong the emergence of resistance. 11 questions representing future challenges in the field of adaptive therapy are shown, and answered within the text. Questions are color-coded by section: integrating the appropriate components into mathematical models (blue), design and validation of dosing protocols (red), and challenges and opportunities in clinical translation (yellow).

In contrast to the periodic administration of dosing under intermittent therapy (e.g., in prostate [Hussain et al., 2013; Crook et al., 2012] or melanoma [Algazi et al., 2020] cancers), adaptive therapy is characterized by dynamic treatment protocols which update (or ‘adapt’) in anticipation to evolving tumor dynamics. These protocols are patient-specific, leading to variable dose modulation or dose timing between patients. While the term adaptive therapy is broad and encompasses many possible dynamic treatment protocols, the term is often used with specific reference to a recent pilot clinical trial in prostate cancer. This first adaptive trial enrolled a small cohort of metastatic castrate-resistant prostate cancer patients, contingent upon a minimum of 50% drop in the level of prostate-specific antigen biomarker (PSA; a proxy for tumor burden) under abiraterone administration. Abiraterone is then withdrawn until PSA returns to pre-treatment levels and then restarted. This 50% rule leads to treatment holidays that are patient-specific (treatment protocol varies considerably between patients). Holidays are often shorter in later treatment cycles when PSA dynamics speed up (Zhang et al., 2017). Initial results of the trial indicate a prolonged progression-free survival and lower cumulative dose when compared to a contemporaneous cohort of patients receiving the standard of care (Zhang et al., 2021). A schematic of adaptive therapy is shown in Figure 1 (purple), which prolongs relapse when compared to a high-dose schedule (blue). While initial results appear promising, this trial was performed on a small cohort of men and did not include a randomized control arm (Mistry, 2021; Zhang et al., 2021). A similar and larger, randomized trial in metastatic castrate-resistant prostate cancer is planned (ANZadapt; NCT05393791).

The first trial has created interest in designing new adaptive treatment protocols in prostate cancer as well as other cancers. Adaptive treatment protocols are often binned into two dose-scheduling approaches: dose modulation and dose skipping. Both are designed to prolong sensitivity to therapy and both have been tested experimentally (Gatenby et al., 2009b; Enriquez-Navas et al., 2016), while only dose skipping has been translated to the clinic (Zhang et al., 2017). Throughout the text, we refer to the following treatment scheduling protocols:

1. Maximum tolerable dose (MTD): periodic administration of a high dose, limited by toxic side effects.
2. Intermittent therapy: periodic administration of a high dose with fixed, periodic treatment holidays.
3. Adaptive therapy (dose skipping): adaptive dosing where a high dose is administered until a desired tumor response (e.g., 50% size reduction), followed by a treatment holiday until a desired upper threshold (e.g., 100%) and repeated.
4. Adaptive therapy (dose modulation): adaptive dosing where dose is modulated (increased or decreased) at regular intervals depending on tumor response.

In Figure 1, we introduce 11 open questions regarding future directions of mathematical modeling in adaptive cancer therapy. These were the result of a 4-d workshop on Cancer Adaptive Therapy Models (CATMo; https://catmo2020.org/) in December 2020. The conference brought together a multidisciplinary group of mathematicians, clinical oncologists, and experimental biologists to discuss successes, challenges and opportunities in adaptive therapy. We have categorized these questions into three sections: (1) integrating the appropriate components into mathematical models, (2) the design and validation of dosing protocols, and (3) challenges and opportunities in clinical translation.

### Integrating the appropriate components into mathematical models

#### What components are necessary for a mathematical model of adaptive therapy?

*Fred Adler*: It is thought that the success of adaptive therapy in delaying the emergence of resistance depends on three characteristics of the cancer: (a) resistance is costly, (b) resistant cells can be suppressed by competition with sensitive cells, and (c) therapy reduces the population of sensitive cells. Simple models based on these assumptions show that adaptive therapy can indeed delay the emergence of resistance. These simple models raise two further questions: (1) What are the appropriate objectives for evaluating the success of therapy? (2) Do the main results hold up in models that include additional components of real tumors?

Our ultimate objective is to maximize survivorship or quality-of-life adjusted survivorship of the patient. This depends on the cancer burden, the treatment burden, and the effectiveness of treatment in suppressing the cancer in the long run (Bayer et al., 2022). In most cases, we do not have sufficient information to quantify each of these costs and benefits over the long run, but we can consider them in concert to evaluate overall success.

Models of adaptive therapy typically include distinct sensitive and resistant cancer cell populations, although some recent models follow a continuum of cell types (Pressley et al., 2021). Model extensions include (a) healthy cells: these cells are always present within a tumor and they will interact with cancer cells (West et al., 2018). (b) Immune cells: these cells can help control cancer but can themselves be affected by treatments (Piretto et al., 2018; Schättler et al., 2016; Park et al., 2019). (c) Resources: hormones (Kareva and Brown, 2021) introduce delays and can alter evolutionary trajectories, and have been modeled as consumer-resource dynamics (Zazoua and Wang, 2019) and more mechanistic models with androgen dynamics (Jain et al., 2011). (d) Allee effect: cell populations that grow more slowly (per capita) at low populations (Konstorum et al., 2016) effectively introduce an element of cooperation. (e) Phenotypic plasticity: rapid changes in cell phenotypes can generate resistance far more quickly than mutation or population dynamics (Salgia and Kulkarni, 2018). See the table below for a list of common modeling approaches used in adaptive therapy.

A key result from our work on the basic model is a tradeoff curve between time for resistant cells to emerge and the mean cancer burden (Buhler et al., 2021). This tradeoff holds for both adaptive (dose skipping) and intermittent therapies, and is robust across all model extensions except for the Allee effect and cell plasticity. With an Allee effect, results are quite different. Recent experimental evidence suggests the presence of an Allee effect in vitro (Johnson et al., 2019), but the extent of this effect is unknown in vivo. Aggressive therapy can drive cells below the threshold and prevent both resistant cells and total cells from reaching their upper thresholds. Adaptive therapy, by backing off early to avoid favoring resistance, can behave quite poorly, leading to escape times nearly as short as those with no therapy and with a high total cell burden.

With the exception of the success of high-dose therapy with a strong Allee effect, no universal therapy can achieve all three objectives of lowering average dose, delaying time to emergence of resistant cells, and reducing total tumor burden. All strategies show a tradeoff between delaying emergence of resistant cells and a high cancer burden. Choosing the appropriate treatment requires assessing the individual patients and specific cancers, and include factors often not included in models, such as therapy toxicity (Ballesta and Clairambault, 2014). Phenotypic plasticity, where resistance is induced by therapy rather than arising from mutations or preexisting variants (Salgia and Kulkarni, 2018), makes resistance much more difficult to suppress (Feizabadi, 2017). Reversible behaviors can create complex responses to therapeutic timing (Hirata et al., 2010).

Effective adaptive therapies require fitting data on individual patients, and data may lack the resolution to distinguish among alternative models. In the case of PSA in prostate cancer, a simple model (Hirata et al., 2010), a more complex model with basic androgen dynamics (Portz et al., 2012), and a detailed model of androgen dynamics (Jain et al., 2011) all fit data on a set of patients reasonably well, although with some exceptions (Hatano et al., 2015). If models can be fit to the dynamics, adaptive therapies may be more robust to patient variability than prescribed timing of intermittent therapy. Although data may lack the resolution to identify specific mechanisms of interaction, such as the strength of competition between different cancer cell phenotypes, simple models may have the greatest potential to capture dynamics and guide therapy. The ideal combination will be patient-specific models combined with in vivo data, perhaps with immunocompetent mouse models, mouse PDX models (Siolas and Hannon, 2013), or in vitro data on patient derived cells that can reveal mechanisms of interaction in different treatment environments.

**Table:** 

Paper	Model type	Key result
Martin et al., 1992b	Gompertzian, Lotka-Volterra	Theoretical models of extension of survival time using dose reduction strategies
Martin et al., 1992a	Gompertzian, Lotka-Volterra	Optimal control methods used to simulate tumors with drug-resistant and drug-sensitive cells in competition
Monro and Gaffney, 2009	Gompertzian	Sensitive-resistant competition can extend survival times, but failed cure can reduce survival
Gatenby et al., 2009b	Mathematical catastrophe theory	The term ‘adaptive therapy’ coined: mathematical and experimental exploration of adaptive therapy
Bacevic et al., 2017	Hybrid cellular automaton	Validation of cost of resistance in CDK inhibitors and role of spatial competition on fitness
Gallaher et al., 2018,	Off-lattice agent-based	Validation of cost of resistance in doxorubicin, and exploration of alternative adaptive protocols
Silva et al., 2012	Frequency-dependent competition	Low doses of auxiliary treatment to accentuate cost of chemo-resistance and improve adaptive therapy
Zhang et al., 2017	Lotka-Volterra	Publication of adaptive prostate trial data and associated Lotka-Volterra competition mathematical model
Smalley et al., 2019	Lotka-Volterra with phenotypic switching	In vivo adaptive dosing of BRAF inhibitors, compared to continuous or fixed intermittent therapy
Kim et al., 2021	Lotka-Volterra with phenotypic switching	Patient-specific prediction of adaptive melanoma therapy
Brady-Nicholls et al., 2021	Density-dependent competition	Patient-specific predictions of adaptive prostate therapy
Strobl et al., 2020	Lotka-Volterra with turnover	Mathematical investigation of cost, turnover, and competition in adaptive therapy
West et al., 2020	Lotka-Volterra	Mathematical investigation of multi-drug adaptive therapy protocols
Viossat and Noble, 2021	Frequency, density-dependent competition	Extensive mathematical analysis of conditions where tumor containment is superior

#### How competitive are treatment-resistant phenotypes?

*Rob Noble*: Adaptive therapy aims to exploit competition between treatment-sensitive and resistant cells. Key questions remain largely unanswered. First, what is the nature of this competition? Mathematical modelers typically assume that the fitness of resistant cells is a simple function of their relative abundance and/or the total tumor size (reviewed in Viossat and Noble, 2021). But frequency- or density-dependent mathematical functions only approximate average population dynamics. Actual clonal growth rates depend on the spatial arrangement of cells, their interaction ranges, and local levels of shared resources, all of which vary both within and between tumors (Noble et al., 2022; West et al., 2021; Fu et al., 2022). For example, if a tumor grows mainly at its boundary then spatial constraints alone could suffice to contain rare resistant clones, but only if they are located away from the boundary (Gallaher et al., 2018; Bacevic et al., 2017). A corollary is that the effectiveness of adaptive therapy may vary between cancer types due to different tumor architectures (Noble et al., 2022). Although spatially structured computational and experimental models can account for some important factors – such as competition for space and oxygen – the ability to predict clinical outcomes hinges on these models accurately matching the parameters of human intra-tumor cell–cell interactions, which remain largely uncharacterized. Further experimental studies and clinical image analyses are needed to quantify these parameters.

Second, are resistant cells less competitive? The seminal 2009 paper by Gatenby et al., 2009b postulated that cells insensitive to therapy incur a fitness cost in the absence of treatment, which adaptive therapy can exploit. A reduction in cell proliferation rate or carrying capacity (defined as the maximal cell density or the maximal cell number in the whole tumor) might result from cells diverting resources away from proliferation and towards breaking down or pumping out toxins. On the other hand, it is uncertain what fitness effects, if any, should result from mutations that modify specific drug targets. Experimental evidence is mixed. A study of tumor containment using a cyclin-dependent kinase inhibitor found a cost of resistance both in vitro and in mice (Bacevic et al., 2017). Conversely, cancer cells resistant to the tyrosine kinase inhibitor alectinib have been observed outcompeting ancestral cells in co-culture (Kaznatcheev et al., 2019). Competition assays should in any case be interpreted with caution because there are many potential mechanisms of resistance to a given treatment and the relative fitness of each phenotype will vary with its microenvironment. Theoretical analyses show that costs of resistance are not necessary to make adaptive therapy superior to higher dose treatment (Strobl et al., 2020; Viossat and Noble, 2021). Nevertheless, such costs – which could be exacerbated by auxiliary treatments (Silva and Gatenby, 2010; Silva et al., 2012) – are typically predicted to amplify clinical gains (Viossat and Noble, 2021).

Lastly, is competition the only important ecological interaction? Studies in vitro and in mice have detected positive ecological interactions (mutualism and commensalism; reviewed in Tabassum and Polyak, 2015) and asymmetric interactions (parasitism) between cancer clones (Miller et al., 1988; Noble et al., 2021). These observations suggest that our theoretical models of clonal dynamics during cancer treatment may be overly simplistic, and they underscore the need for more and better data. Emerging spatial genomic, transcriptomic, and proteomic technologies (Seferbekova et al., 2022) hold particular promise for inferring subclonal interactions within human tumors. Below, several sections discuss challenges and opportunities integrating mathematical models with wet lab data (How can we leverage mathematical modeling to support testing of adaptive therapy in the wet lab?) and clinical data (Is real-time patient prediction feasible?).

#### What is the role of plasticity and drug-induced mutations in adaptive therapy?

*Eunjung Kim*: The effectiveness of treatment holidays drastically changes when considering phenotype switching between drug-sensitive and -resistant phenotypes (Pillai et al., 2022). Plasticity is often modeled as the expression of resistant cellular traits that vary from completely sensitive to fully resistant (Clairambault and Pouchol, 2019; Clairambault, 2019), in multidimensional fashion to consider multi-drug resistance (Cho and Levy, 2018a; Cho and Levy, 2018b). Treatment breaks can halt the expansion of the resistant cell population facilitated by drug-induced mutations or phenotype switching from sensitive to resistant states during therapy. Since phenotype switching to resistant states is often reversible (reviewed in Boumahdi and de Sauvage, 2020), treatment holidays have the potential to re-sensitize the resistant cell population to future drug rechallenges. A recent experimental study demonstrated that gene expression in melanoma cells reversed during treatment holidays, causing the cells to re-sensitize to a BRAF inhibitor rechallenge (Kavran et al., 2022).

The switching rate from resistant to sensitive states can impact the benefit of adaptive therapy. One recent study introduced a mathematical model of plasticity-mediated drug resistance of melanoma treated with BRAF/MEK inhibitors (Hodgkinson et al., 2022). Here, the mathematical modeling predicts small differences resistance emergence between continuous and adaptive therapy, but the latter leads to increased spatial heterogeneity. Another recent study integrated mathematical models with clinical data of a cohort of patients with melanoma treated with continuous therapy of BRAF/MEK inhibitors (Kim et al., 2021). The resulting calibrated mathematical models then simulated alternative treatment protocols. Modeling predicted that adaptive therapy (dose skipping) outperforms standard of care at different degrees among the patients (Kim et al., 2021). Among mathematical model parameters that govern treatment response dynamics, both the switching rate from resistant to sensitive states and the growth rate of sensitive cells determine the benefits of adaptive therapy. In another mathematical modeling study, a fixed schedule intermittent therapy was predicted to outperform the standard of care when treatment could induce resistant mutations in the cells (Greene et al., 2019). These properties of tumor plasticity or drug-induced mutation are variable between cancer types and possibly vary between cancer cells. For example, in melanoma, it was shown that phenotypic plasticity is more evident in one cell line than another (Smalley et al., 2019). There may be even more variability across patients in terms of how resistance emerges and is maintained. Thus, identifying the presence of phenotypic plasticity in a specific tumor could be an important factor in deciding if and how adaptive therapy should be applied.

#### What is the role of homeostasis and normal tissue in adaptive therapy?

**Figure 2.**
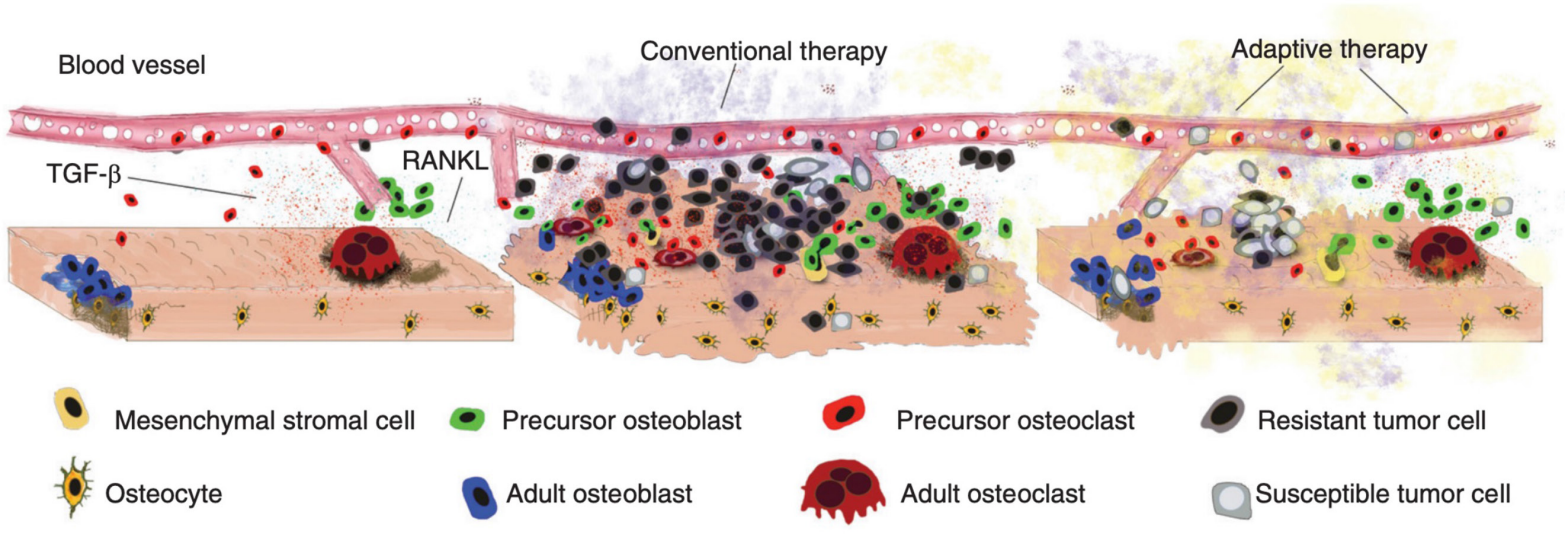
Disruption and restoration of tissue homeostasis. Left: bone tissue homeostasis, including bone resorption by osteoclasts and osteoblasts. Middle: tumor cells cause disruption of homeostasis, leading to altered microenvironment factors. Conventional therapy leads to increasing tumor resistance. Right: evolution-based treatment strategies aim to restore some degree of homeostasis while allowing the tumor to remain sensitive to future treatment.

*David Basanta*: A feature of current models of adaptive therapy lies in their simplicity in terms of algorithms and assumptions. One key simplification is that tumor heterogeneity can be reduced to the types of cancer cells such as sensitive cells (that pay a significant fitness cost during treatment) and treatment-resistant cells (that may incur a cost of resistance relative to sensitive cells). In reality, the fitness of a cancer cell is not simply a cell-intrinsic property but includes its ability to take advantage of its dynamic tumor environment that includes not just other cancer cells but normal cells, vasculature, immune cells, and extracellular matrices (see Figure 2).

Far from living in isolation, cancer cells colonize tissues with an existing ecosystem made up of healthy, stromal cells that communicate with each other via molecular factors in order to maintain homeostasis. The tissue has a distinct physical and spatial architecture (Basanta and Anderson, 2013). In the bone, for instance, homeostasis results from the interactions between several cell types such as osteoblasts, osteoclasts, osteocytes, monocytes, macrophages, and mesenchymal stem cells (MSC) (Bussard et al., 2008). While metastasis is a highly inefficient process, successful colonization of the bone by metastasizing prostate cancer cells leads to a process called the vicious cycle (Esposito et al., 2018; Cook et al., 2014). Successful prostate cancer cells in the bone take advantage of the interactions and signaling that goes on between the normal cells as they maintain homeostatic tissue microenvironments (Basanta and Anderson, 2013). Factors released by normal cells such as transforming growth factor β are utilized by nearby prostate cancer cells. Such factors accelerate the proliferation and survival of the cancer cells.

Proximity to stromal cells provides other benefits to cancer cells undergoing treatment. For instance, bone metastatic prostate cancer cells near MSCs are pre-selected to possess some level of chemoresistance (McGuire et al., 2021). Such cells are primed for resistance even prior to treatment exposure. Also in the bone, myeloma cells (bone cancer) near MSCs or in the presence of growth factors secreted during bone remodeling can survive standard of care treatments based on proteosome inhibitors like bortezomib (Xu et al., 2012). This environmentally mediated drug resistance (EMDR) explains why anticancer treatments prove less effective than otherwise expected. In the context of adaptive therapy, EMDR may provide the cancer cells with a therapy refuge regardless of whether they are resistant or sensitive to treatment.

For cancers where EMDR plays a large role, it may be advisable to be more aggressive in applying therapy and reducing the tumor burden. This is because treatment sensitive cells will remain viable even after large reductions in tumor burden. These sensitive cells surviving in or near a stromal refuge can then provide a source of competition for resistant cancer cells during drug holidays. Furthermore, one can include adjuvant treatments, targeting stromal cells, to modulate the role of EMDR. The goal of EMDR modulating drugs would not be to maximize tumor kill. Rather, such modulation would aim to improve the efficacy of adaptive therapy approaches to better control tumor burden and maintain quality of life over long periods of time.

Future adaptive therapy models should allow for EMDR. Such models could more faithfully incorporate the tumor microenvironment and the role of normal cells. Such models could then evaluate how best to manage or exploit EMDR when designing adaptive therapy protocols (M A et al., 2022). Even more might be gained by developing models that also recapitulate tissue homeostasis prior to carcinogenesis. As discussed elsewhere (Basanta and Anderson, 2017), cancers initiate in normal tissue environments and progressively overcome and exploit the rules of homeostasis. Adaptive therapy aims to control the tumor by introducing a different type of homeostasis. Hence, in improving the original adaptive therapy algorithm, we should consider the homeostasis that was disrupted by the tumor as well as the homeostasis that might be engineered by therapy.

### Design and validation of dosing protocols

#### Cure or control?

*Jill Gallaher*: An explicit goal of adaptive therapy is to turn cancer into a chronic disease with sporadic (but life-long) management. Taking this approach likely means abandoning the hope of cure. Thus, its current appeal and modeling contexts have been for patients with essentially no curative options. However, there are cases when adaptive therapy would have been preferred when standard of care results in recurrence and adaptive therapy would have either resulted in longer control or less dose and therefore better quality of life. In other cases, standard of care could result in cure or longer control. But is identifying such patients prior to treatment even possible? Furthermore, if the standard of care regimen is tried and cure does not result, then it may not be possible to switch to an adaptive therapy regimen because at that point the resistant population of cancer cells may be too large compared to the sensitive cells to establish sufficient control (McClatchy et al., 2020). The window for extended disease control using an adaptive therapy protocol may only be open prior to treatment. The decision must be made at the start. If one only opts for standard of care for cure, failed curative attempts could lead to reduced survival times (Monro and Gaffney, 2009) relative to adaptive therapy. So how does one decide between these opposing strategies? What key disease characteristics are needed to stratify patients into a treat-to-cure cohort (using continuous therapies) versus a treat-to-contain cohort (using adaptive therapies) (Hansen and Read, 2020a)?

Towards deciding between treat-to-cure or treat-to-contain strategies, bacterial studies in addition to cancer studies provide some insights. Initial pathogen heterogeneity may favor adaptive therapies. The treatment response of heterogeneous bacterial colonies depends on competition and tradeoffs among the bacterial strains. Colijn et al. studied how competition between microbial populations affects the optimal dosing of antibiotics (Colijn and Cohen, 2015). Large antibiotic doses were in some cases observed to reduce the bacterial load and prevent resistance and in other cases to select for more antibiotic resistant cells. They found that even if aggressive treatment was optimal for individual strains, moderate treatments were better to avoid resistance for the entire community when there was strong inter-strain competition (Colijn and Cohen, 2015). Hansen et al. proposed a balance threshold hypothesizing that containment delays progression only if the overall effect of competitive suppression exceeds the overall effect of mutational input (Hansen and Read, 2020a; Hansen et al., 2017). In cancer, like bacteria, tradeoffs between therapy resistance and competitiveness in the absence of therapy favor adaptive therapies over treat-to-cure. But these tradeoffs among cancer cells may only manifest in specific contexts or through temporary constraints on cell functions (Strobl et al., 2020). Furthermore, the effect of competition and tradeoffs may lessen if cells are not in direct contact (Strobl et al., 2022). For example, if the tumor is very invasive or if different metastases have different compositions of sensitive and resistant cells, the effects of tradeoffs and competition are reduced (Gallaher et al., 2018; Gallaher et al., 2022). Diverse metastatic sites or spatial segregation of heterogeneous cancer cells within tumors mean that different tumors or locations within tumors may respond strongly to treatment while others grow unimpeded. In these cases, an attempt at cure might be better than containment (Gallaher et al., 2022).

The ratio of sensitive to resistant subpopulations as well as the transition rates between them influence the efficacy of adaptive therapy and continuous treat-to-cure therapies. Both benefit from there being a low frequency of resistant cancer cells. But adaptive therapy may benefit more. An aggressive, maximum-tolerated chemotherapy approach will result in a larger initial response to therapy (Hansen and Read, 2020b), but if there is not complete disease eradication, even a small or emergent population of resistant cancer cells can guarantee eventual disease progression. High doses necessarily promote the competitive release of preexisting resistant cells. However, high doses may also prevent de novo drug resistance by lessening the pool of sensitive cells from which resistant cells emerge (Colijn and Cohen, 2015). Phenotypic plasticity poses another challenge for both therapeutic strategies where the application of treatment accelerates the transition of cancer cells into resistant states (see What is the role of plasticity and drug-induced mutations in adaptive therapy?). Nevertheless, adaptive therapy may be favored when treatment breaks result in the re-sensitization of cancer cells to the drug.

Overall disease burden and initial response to therapy should influence the choice of cure versus containment. In the case of antimicrobial drugs, Kouyos et al. evaluate the decision for aggressive versus moderate dosing in terms of two opposing ecological and evolutionary processes (Kouyos et al., 2014). Ecologically, the rate of disease burden reduction will increase with increasing dose. Evolutionarily, increasing the dose increases the selection pressure for resistant microbes (Kouyos et al., 2014). Their work concludes that the optimal dose should be high enough to reduce the patient’s disease burden, and low enough to forestall the emergence of resistance. Adaptive therapy attempts to break this constraint by applying high doses when the frequency of sensitive cells is high and removing therapy when this frequency has declined. Yet, successful adaptive therapies may mean maintaining large tumor burdens to promote competition. On the other hand, the high tumor burden may increase the likelihood of new metastases or evolutionary breakthroughs by the cancer cells. In addition, the patient must be able to tolerate the large burden without debilitating or life-threatening symptoms.

The tumor burden alone does not reveal the underlying dynamics of cell turnover (Gallaher et al., 2019), which is also important for treatment response. This background turnover results from the replacement through proliferation of cells that regularly die from spontaneous apoptosis, an immune response, or lack of resources. Both therapeutic strategies can benefit from a higher background cell turnover rate. An aggressive treatment may substantially decrease the tumor burden and increase the probability of cure by increasing the chance of spontaneous death of the resistant population (Strobl et al., 2022). An adaptive therapy approach benefits from a high cell-turnover rate by increasing the competition to mutational input balance threshold (Hansen and Read, 2020a) and increases the opportunities for sensitive cells to replace resistant cancer cells when therapy is off.

There are also practical clinical considerations of using each treatment strategy. An adaptive protocol must have frequent measures on which to base the decisions of when to increase or decrease dose rates. For adaptive therapy in prostate cancer, PSA is used as a surrogate for burden. In other cancers, it may be imaging, ctDNA, or other molecular markers (see Is real-time patient prediction feasible?). The biomarker used for decision-making needs to accurately measure changes in the disease burden and state. Frequent measures are best, thus inexpensive and less invasive biomarkers are favored. Ideally, decisions for adaptive protocols could also be guided by measurements of drug resistance, evolvability, or competition if possible. Otherwise, these measurements might be used as stratification factors from pretreatment tissue biopsy. A short induction period to determine disease kinetics could help with the stratification of patients with higher or lower likelihoods of cure under an aggressive treatment strategy. This allows for some measures of the cancer’s eco-evolutionary dynamics without committing to a specific therapeutic regimen (McClatchy et al., 2020). Further, overall survival is an important measure for comparing treatment strategies, but it must be balanced with toxicity and quality of life (Milano et al., 2021). For successful adaptive therapy, drug timing, which includes pharmacokinetics and pharmacodynamics, must be aligned with the growth rate of the tumor and the accumulating side effects for the patient. Attempting to cure a tumor with a slow response means a longer application of aggressive treatment, so drug toxicity becomes a key consideration. With adaptive therapy, the treatment breaks can improve quality of life and reduce overall dose rates, but there is potential for accumulating side effects over an indefinite course of therapy. Cure or control could be favored depending on the patient, the disease state, and the drugs used.

#### What is the optimal adaptive dose administration protocol?

*Yannick Viossat*: Adaptive therapy often refers to the specific protocol used in the initial prostate clinical trial (Zhang et al., 2017). However, the concept has wider applicability (Gatenby et al., 2009b; Enriquez-Navas et al., 2016; Bacevic et al., 2017; Carrère, 2017; Gallaher et al., 2018; Viossat and Noble, 2021; Cunningham et al., 2018; Cunningham et al., 2020; Hansen and Read, 2020b). The prostate trial’s design was driven by a compromise between mathematical model results and clinically feasible treatment protocols. In this section, we review optimal protocols revealed through investigations of mathematical models. Subsequent sections review the best practices to incorporate experimental (How can we leverage mathematical modeling to support testing of adaptive therapy in the wet lab?) and clinical data (Is real-time patient prediction feasible?) relating to dose modulation protocols.

Many mathematical models emphasize competition between sensitive and highly resistant cells and assume that the larger the tumor size, the stronger the competition (Martin et al., 1992b; Monro and Gaffney, 2009; Zhang et al., 2017; Carrère, 2017; Carrère and Zidani, 2020; Martin et al., 1992a; Strobl et al., 2020; Viossat and Noble, 2021). Such models suggest maintaining the tumor at the maximal acceptable size in order to maximize competitive suppression of resistance (Hansen and Read, 2020b; Viossat and Noble, 2021). This may require delaying treatment if the tumor is initially small (Monro and Gaffney, 2009; Cunningham et al., 2020; Hansen and Read, 2020b; Viossat and Noble, 2021). Time to tumor progression may be delayed by switching to high doses a short time before containment fails (Wang et al., 2021b; Viossat and Noble, 2021), but at the risk of making the tumor less treatable afterwards (Viossat and Noble, 2021). Moreover, aggressive treatment may increase toxicity and drug-induced mutations (Kuosmanen et al., 2021). However, maintaining a substantial tumor burden may create other potential problems: a lower quality of life, more metastases, increased mutation from sensitive to resistant tumor cells (Martin et al., 1992b; Hansen et al., 2017), or the appearance of new tumor cell types. Some models with a death rate that increases proportionally with tumor size find that MTD is superior to adaptive therapy (dose skipping) (Mistry, 2020).

If resistant cells are only partially resistant, they may be targeted by treating mildly, to exploit competition with sensitive cells, or aggressively, to exploit their remaining sensitivity. Theoretical models suggest that a sensible strategy is to first exploit competition by stabilizing tumor size, but then switch to MTD long before stabilization fails, as opposed to stabilizing the tumor for as long as possible when resistant cells are fully resistant (Hansen and Read, 2020b; Viossat and Noble, 2021). The switching time could be timed with a decrease of treatment efficiency (Wang et al., 2021b), but the practical implementation and whether similar conclusions hold for models with many types of tumor cells (Pouchol et al., 2018) remains to be investigated.

Mathematical modeling has extensively explored differences between dose-modulation or dose-skipping (Enriquez-Navas et al., 2016; Gallaher et al., 2018). It is important to note there are several clinical case studies of intermittent therapy that lead to significant prolonging of time to progression (e.g., androgen deprivation therapy in prostate cancer [Matsuzaki et al., 2019], and carboplatin and docetaxel chemotherapy in metastatic lung adenocarcinoma [Shida et al., 2017]). This illustrates both the feasibility and the potential promise of treatment holidays. These clinical reports must be integrated into theoretical frameworks. For example, if cell–cell competition increases with tumor size, a continuous low-dose treatment maintaining the tumor at a given threshold may be preferable to an intermittent treatment maintaining it between this threshold and some lower size. This is not a compelling argument against intermittent treatments though: indeed, the same models suggest that an intermittent treatment maintaining tumor size between this threshold and some larger size may increase competition even more (Hansen and Read, 2020b; Viossat and Noble, 2021).

As referenced in the introduction, initial clinical trials (e.g., NCT02415621) employ a ‘rule of thumb’ decision for treatment holidays, such as the 50% rule. Mathematical modeling has investigated alternative simple rules, such as range-bounded adaptive therapy (Brady-Nicholls and Enderling, 2022), which is designed to increase competitive inhibition of resistant subpopulations with a straightforward clinical implementation (Hansen and Read, 2020b).

It has been hypothesized that tumor stabilization might also normalize tumor vasculature, leading to a larger drug efficiency (Gatenby et al., 2009b; Enriquez-Navas et al., 2016), and dose-modulation might normalize tumor environment more than dose-skipping (Enriquez-Navas et al., 2016). Evidence remains scarce. In a mouse model (Enriquez-Navas et al., 2016), a dose-modulation strategy was more effective than a dose-skipping strategy, but maybe because the cumulative dose in the dose-modulation arm was higher.

In theoretical models, a tumor may be temporarily stabilized by a constant dose treatment during a short time interval. A practical question is to determine the appropriate stabilization dose. Cunningham et al., 2020 found that an upward dose-titration protocol, gradually increasing the dose until the tumor is stabilized, works better than a dose reduction protocol (see also Masud et al., 2022 for calculating an effective dose window). Indeed, starting from a high dose may quickly select for resistant phenotypes. However, with upward titration, tumor size may become dangerously large before the dose is sufficiently high to stabilize it. Rather than a fixed dose modulation (e.g., 10%), weighting the dose according to tumor responsiveness may lead to quicker stabilization (Viossat and Noble, 2021). Moreover, some protocols keep the same dose if tumor size changes little since the previous measurement (Gatenby et al., 2009b; Enriquez-Navas et al., 2016; Gallaher et al., 2018). Successive small changes, with a large total effect, may then never trigger dose-modulation. This suggests the next dose should depend on the most recent change in tumor size but perhaps also on its absolute size compared to some target (Viossat and Noble, 2021). Many of these ideas remain to be empirically tested and may be difficult to implement clinically.

Finally, agent-based models (You et al., 2017; Bacevic et al., 2017; Gallaher et al., 2018; Strobl et al., 2022) allow for testing of features that are not easily incorporated into differential equation models: spatial structure, cell mobility, or quiescence. Spatial structure may increase the cost of resistance, as resistant cells may be trapped inside the tumor, far from the proliferative edge. These models also lead to observations that are not easy to understand theoretically, such as the greater efficiency of dose-skipping over dose-modulation in Gallaher et al., 2018. This highlights that simple models may miss important phenomena and that more data and modeling are needed to optimize adaptive therapies.

#### How can we leverage mathematical modeling to support testing of adaptive therapy in the wet lab?

*Maximilian Strobl*: Thanks to promising preclinical and clinical results, there is growing interest in extending adaptive therapy to new disease settings. To do so requires experimental platforms for testing and, if necessary, improving the safety and efficacy of adaptive protocols. Experimental systems are models and come with inherent assumptions and limitations. Mathematical modelers and experimentalists should collaborate closely in order to design preclinical studies to validate theoretical models, assert safety, and develop adaptive protocols with the maximum benefit to patients.

Surveying the experimental literature on adaptive therapy, and based on our own experience, we identify three areas in need of further research. First, how do we design experiments to assess the competitive suppression in a particular cancer and thus the scope for such patients to benefit from adaptive therapy? To date, experiments have employed one of three model systems, or combinations thereof: (i) 2-D in vitro cell culture (e.g., Silva et al., 2012; Bacevic et al., 2017; Farrokhian et al., 2022; Nam et al., 2021; Bondarenko et al., 2021), (ii) 3-D in vitro spheroids (e.g., Bacevic et al., 2017; Strobl et al., 2020; Bondarenko et al., 2021), and (iii) subcutaneous in vivo mouse models, in which human cells are injected into immunocompromised animals (e.g., Gatenby et al., 2009b; Enriquez-Navas et al., 2016; Smalley et al., 2019; Wang et al., 2021b; Wang et al., 2021a). 2-D and 3-D in vitro models are inexpensive and quick, and allow for easy manipulation and monitoring of the ‘tumor.’ In contrast, by incorporating vasculature and stroma, orthotopic mouse models are more realistic, but they are expensive. Mouse models often do not include an immune system and see human cells competing with mouse rather than human cells. A solution to this problem will be the use of more advanced technology, such as organ-on-chip models (Kashaninejad et al., 2016; Wang et al., 2021c) or spontaneous mouse models, where mouse tumors develop ‘naturally’ in their tissues of origin (Kersten et al., 2017; Céspedes et al., 2006). But even with more advanced experimental systems, limitations remain. To address these, we need to better understand what cells compete for (as discussed in section 1.2), and how we can best quantify this competition (e.g., the ‘game assay’; Kaznatcheev et al., 2019; Farrokhian et al., 2022). We propose that by playing out different scenarios in silico, mathematical models can help us to refine what experiments we should perform, and in what experimental system(s), in order to deduce the competitive landscape in tumors and in order to inform on how adaptive therapy will perform in patients.

Second, there is the question of how drug resistance is modeled in the wet lab. One approach evolves resistant cells through long-term drug exposure, and subsequently performs experiments by mixing these cells with parental, sensitive cells (e.g., Bacevic et al., 2017; Nam et al., 2021; Wang et al., 2021b). This has the advantage that the resistant population can be characterized (e.g., measuring its growth rate), the initial resistant cell fraction can be controlled, and cell populations can be fluorescently tagged and followed over the course of the experiment. However, this design implicitly assumes the preexistence of resistant cells in the tumor and neglects the role of plasticity. Alternatively, drug resistance can be allowed to evolve naturally over the course of the experiment (e.g., Gatenby et al., 2009b; Enriquez-Navas et al., 2016; Smalley et al., 2019). But such experiments are time consuming and provide less information about the resistant population. We suggest using mathematical modeling to examine how best to leverage each of these two approaches to gain information on the cancer of interest. In addition, emerging clone-tracking technology allows for ever more in-depth study of tumor evolution (Morgan et al., 2021). Mathematical modeling will be a useful tool in designing, and interpreting results from, clone-tracking experiments (Acar et al., 2020; Damaghi et al., 2021; Johnson et al., 2020).

Finally, there is the question of how to translate treatment algorithms from mathematical or experimental models into clinical practice. Most mathematical models of adaptive therapy neglect drug pharmacokinetics, but clearly this impacts the drug delivery to the tumor and differs between animals and patients. In addition, there is a question of time scales: how does a weekly follow-up in mice compare to a reassessment every 3 mo in patients? And, what happens when treatment cannot be adjusted as planned due to toxicity or practical constraints (e.g., machine failure, or the intended day falling on a holiday/weekend)? This raises the question of how robust are adaptive schedules to deviations, and what is the best strategy with which to respond when deviations occur. Some initial work on this topic has been carried out (Dua et al., 2021; Wang et al., 2021a), and we encourage more research in this direction in order to inform experimental and clinical trial design.

#### What are the best practices to design adaptive algorithms for multiple drugs?

*Jeffrey West*: It remains unclear how to extend adaptive therapy approaches to multiple treatments. When multiple drugs are available, the combinatorial possibilities expand rapidly. With n treatments available, there exist $2^{n}$ possible combinations, each of which may be administered at each treatment decision point. Current adaptive trials often utilize less than the full range of $2^{n}$ combinations. For example, the metastatic castrate-resistant prostate cancer adaptive trial (NCT02415621) administers Lupron (Leuprorelin; gonadotropin-releasing hormone analogue for medical castration) as a continuous backbone while Abiraterone acetate (an inhibitor of CYP17A1, designed to suppress the production of androgens) is given adaptively. The advanced BRAF-mutant melanoma adaptive trial (NCT03543969) administers Encorafenib (a small molecule BRAF-inhibitor) and Binimetinib (a selective MEK inhibitor) in combination adaptively, with Nivolumab (an immune checkpoint inhibitor that blocks PD-1) administered continuously. In both examples, opening the trial design to include the full range of treatment permutations may extend therapeutic control, but at the cost of computational and investigational complexity.

Recently, the concept of steering tumor dynamics into periodic, repeatable evolutionary cycles was proposed (Newton and Ma, 2019; Ma and Newton, 2021; Liu et al., 2022). The ordering and timing of treatment combinations is chosen carefully to drive tumor phenotypic composition into a ‘cycle’ such that tumor composition at the start and end of a cycle of therapy are approximately equivalent (West et al., 2020; Dua et al., 2021). Evolutionary cycling was implemented as a strategy to combat resistance to osimertinib (a third-generation tyrosine kinase inhibitor) in EGFR-mutant non-small cell lung cancer (Wang et al., 2021a). Dynamics were described by a Lotka-Volterra competition model within a nonlinear mixed-effects modeling framework, and potential treatment schedules were screened in silico to select fixed protocols that drive tumor dynamics into periodic cycles. These fixed treatment plans implemented in vivo outperformed standard of care treatment schedules in a majority of cases. This study and others (Wang et al., 2021b; Thomas et al., 2022; West et al., 2020; West et al., 2019) illustrate the feasibility of model-driven treatment planning to reduce the combinatorial complexity for multi-drug adaptive therapies.

Traditionally, adaptive therapy abandons the goal of cure in favor of long-term control (see Cure or control?). However, the availability of additional treatments affords the opportunity to leverage evolutionary principles for cure. Below, we explore two evolution-based treatment strategies: (1) double-bind therapy and (2) extinction therapy. Strictly speaking, these approaches cannot be classified as ‘adaptive’ but it is important to consider the feasibility and potential promise of these alternative evolution-based strategies.

The first alternative evolution-based multidrug approach is to identify collaterally sensitive treatments such that resistance to first-line treatment induces sensitivity to secondary treatments (Silva and Gatenby, 2010; Basanta et al., 2012). In double-bind therapy, the drugs are given sequentially rather than in combination (Gatenby et al., 2009a). Using mathematical models, it is possible to determine the optimal switching time between a pair of collaterally sensitive drugs (Yoon et al., 2018). This approach can be extended to infer the optimal timing and ordering of a set of collaterally sensitive drugs (Yoon et al., 2021), which can allow for evolutionary steering (Iram et al., 2021; Acar et al., 2020) or even extinction (Gatenby et al., 2020a; Gatenby et al., 2019). A similar strategy termed the ‘primary-secondary’ approach administers the primary treatment adaptively, while the secondary treatment is administered within each adaptive treatment cycle in order to suppress long-term resistance (West et al., 2019).

A second alternative multidrug approach known as extinction therapy may provide a way out of the control versus cure conundrum introduced in Cure or control?. For many incurable cancers or specific patients that failed to be cured, an aggressive therapy given continuously will generate a complete response rendering the cancer temporarily clinically undetectable sometimes for periods of years, other times for just months. Rather than wait for the period of remission to end before switching therapies, extinction therapy aims to exploit the vulnerabilities of small, fragmented populations (Artzy-Randrup et al., 2021; Johnson et al., 2019; Konstorum et al., 2016). In this case, these small populations are the remnants that survived therapy either by virtue of resistance or position within the tumor (sometime referred to as stromal protection when the structure of normal cells prevent therapy reaching cancer cells). In models of extinction therapy, the initial therapy (called the first-strike) is stopped as soon as the disease burden shows a complete response (Gatenby et al., 2019; Gatenby and Brown, 2020b). At this point, therapy becomes a sequence (e.g., 45–90 d per sequence) of second strikes using different drugs with different modes of action, and that will not generate undue toxicities. While untried, one can imagine starting a patient that might be cured with an aggressive therapy. If this therapy only generates a partial response, then immediately switch to another drug and/or an adaptive therapy before disease progression. If the therapy produces a complete response, then go into an extinction therapy regimen aiming for cure. If permanent remission does not ensue, then the initial first strike drug likely is still effective, and can then be used for an adaptive therapy. By switching therapies sooner before complete resistance has evolved, the physician and patient retain the option for switching to an adaptive therapy. While models of extinction therapy have been developed (Gatenby et al., 2020a), clinical evidence is sparse but supported by the standard of care multistep curative treatment in Pediatric Acute Lymphocytic Leukemia (Li et al., 2022), by two case studies involving cure in patients with metastatic breast cancer (Chue and La Course, 2019a; Chue and La Course, 2019b), and an ongoing clinical trial for patients with pediatric rhabdomyosarcoma (Reed et al., 2020).

### Challenges and opportunities in clinical translation

#### Is real-time patient prediction feasible?

*Renee Brady-Nicholls*: Predicting precisely when a patient will progress during adaptive therapy offers the opportunity to appropriately modulate treatment, thereby extending patient response and survival. This requires sufficient monitoring of an individual patient’s disease using appropriate clinical markers. Choosing an appropriate biomarker depends on the extent of the disease (e.g., localized versus metastatic, or hormone sensitive versus castration resistant prostate cancer), as well as how frequently said biomarker can be collected to adequately follow the disease trajectory.

Prior to making model predictions, the chosen model should be calibrated and validated to demonstrate that it can accurately describe patient-specific biomarker dynamics (Brady and Enderling, 2019). This requires analyzing the model to determine the sensitivity and identifiability of model parameters, relative to the data. Given a chosen model and available data, model parameters may be difficult to accurately estimate. This might be due to the model complexity or structure, as well as the given data. Evaluating the sensitivity of the model outputs, in this case the change in a modeled biomarker over time, with respect to small perturbations in the model parameters identifies sensitive and insensitive parameters (Banks and Tran, 2009). Sensitive parameters should be evaluated for correlations with other parameters as correlated parameters should not be estimated concurrently. If two parameters can be uniquely identified, then they are said to be identifiable (Olufsen and Ottesen, 2013) and parameter optimization techniques can be used to determine their optimal values relative to the given data. Appropriate model development, calibration, and validation are essential when developing predictive models. If the model cannot accurately describe the data, then it should not be used to make predictions of subsequent patient responses.

**Figure 3.**
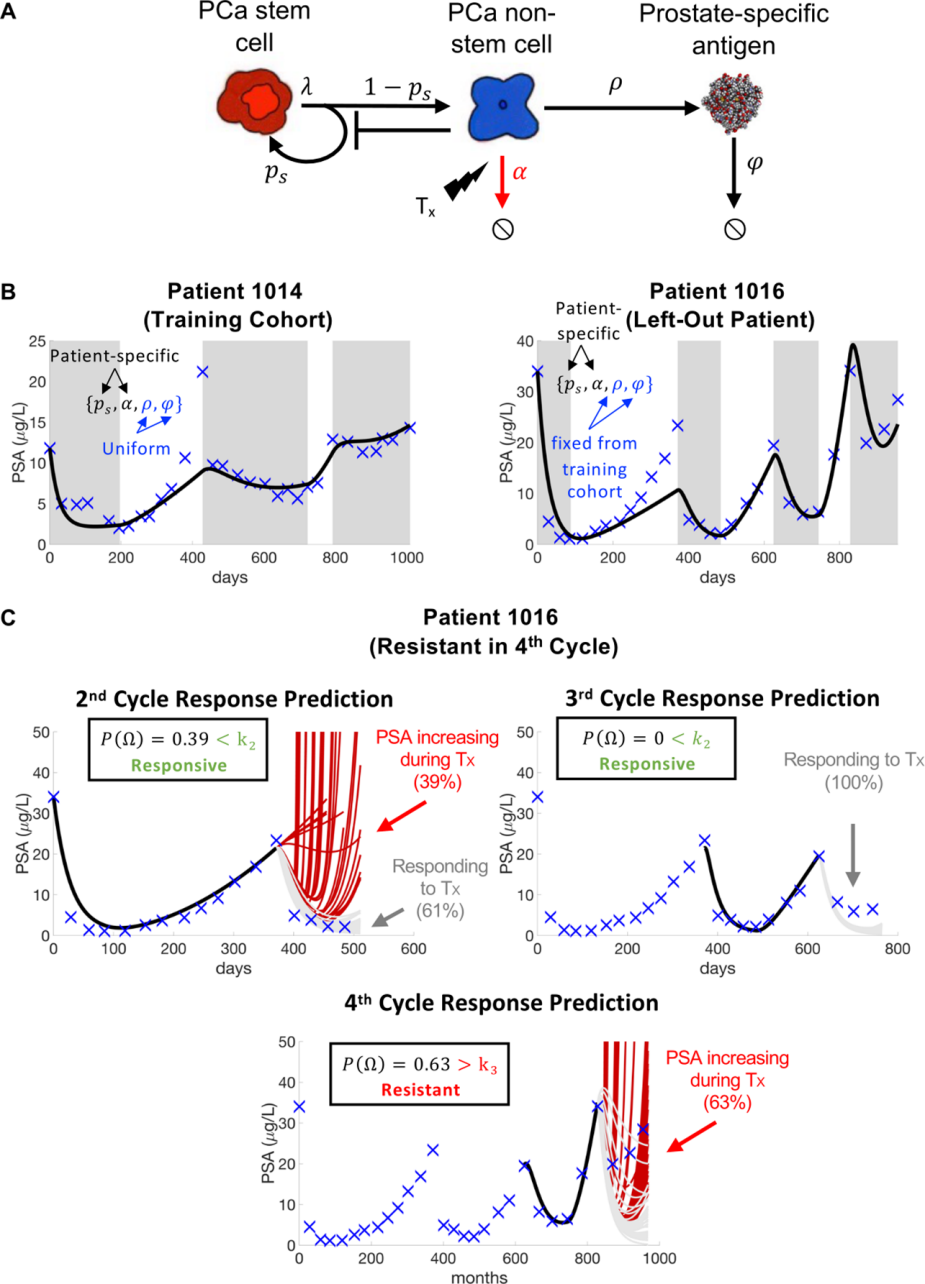
Model schematic, calibration, validation, and prediction. Adapted from Figure 4 of Brady-Nicholls et al., 2021. (**A**) Model schematic of treatment-resistant stem cells, sensitive non-stem cells, and prostate-specific antigen interactions. (**B**) Model calibration (patient 1014) and validation (patient 1016). Nested optimization was used to determine the cohort uniform parameters ρ and φ and the patient-specific parameters $p_{s}$ and α for the training cohort. The uniform values were fixed in the testing cohort, and optimization was used to find the patient-specific parameters $p_{s}$ and α. (**C**) Model predictions for patient 1016. The model predicted resistance in 39% of cycle 2 simulations and response in 100% of cycle 3 simulations. Cycle 4 predictions showed resistance in 63% of model simulations. Using cycle-specific cutoffs $k_{2},k_{3}$, and $k_{4}$, the model correctly predicted that patient 1016 would continue to respond in cycles 2 and 3 but become resistant in cycle 4.

Figure 3 illustrates a case study of the feasibility of real-time patient prediction. Here, the longitudinal biomarker known as prostate-specific antigen (PSA) is used to fit a mathematical model and provide a patient-specific prediction in response to adaptive therapy in metastatic castration-resistant prostate cancer (Brady-Nicholls et al., 2021). Despite much controversy surrounding the clinical use of the absolute value of PSA in both the detection and monitoring of prostate cancer (Kim and Andriole, 2015), it has been shown to be an effective and inexpensive way to follow a patient’s response trajectory over time. Many mathematical models based on a variety of plausible mechanisms have been developed to describe PSA dynamics in response to treatment (Zhang et al., 2017; Baez and Kuang, 2016; Hirata et al., 2010; Morken et al., 2014; Portz et al., 2012).

The mathematical model (Figure 3A) uses stem and non-stem cell dynamics to describe patient-specific PSA dynamics in response to treatment. The model has five parameters ($p_{s}$, λ, α, ρ, φ). Sensitivity analysis found that λ was insensitive, while identifiability analysis showed that $p_{s}$, α, ρ, and φ were uncorrelated and identifiable. A leave-one-out analysis was used to calibrate and validate the model to longitudinal PSA data from 16 patients. That is, nested optimization was used to estimate patient-specific parameters for $p_{s}$ and α, and uniform parameters for φ and ρ to accurately describe individual patient data for the 15 patients in the training cohort. The uniform φ and ρ values were then fixed and optimization was used to find the $p_{s}$ and α values for the left-out patient (Figure 3B). Once calibrated and validated to the patient data, the model was used to make patient-specific response predictions. Parameter analysis identified the stem cell self-renewal rate $p_{s}$ as the primary driver of differences in treatment response dynamics between responsive and resistant patients. This parameter was used to make subsequent response predictions. That is, the distribution of changes in $p_{s}$ from treatment cycle i to i+1 was used to predict an individual patient’s response in cycle i+1 (Figure 3C). The model was able to predict patient response with 81% accuracy (Brady-Nicholls et al., 2021) (defined as the sum of correct responsive and resistant predictions divided by the total number of predictions). A similar modeling approach was used in biochemically recurrent prostate cancer patients receiving intermittent androgen deprivation therapy to predict response dynamics with 89% accuracy (Brady-Nicholls et al., 2020). Clinically, an accurate predictive model can be used an additional tool that oncologists can use when making treatment decisions for individual patients. An accuracy above 50% (coin-toss) can provide clinicians with more confidence when making such critical decisions.

We can learn several lessons from these studies when applying real-time prediction of adaptive therapy in new diseases. Model predictions are dependent on the quality and time-resolution of patient-specific biomarkers. Alternative biomarkers such as circulating tumor DNA (ctDNA) (Hennigan et al., 2019; Ku et al., 2019; Lau et al., 2020), circulating tumor cells (CTCs) (Ried et al., 2020; Salami et al., 2019), and relatively new biomarkers such as urine Lemos et al., 2019; Tosoian et al., 2021 have been shown to be prognostic in prostate cancer and other diseases. Like PSA, these markers can be collected relatively frequently and via minimally invasive methods. They can be used to develop appropriate models that can be used to predict response to adaptive therapy.

#### Do adverse effects of maintaining high tumor burden negate potential benefit?

*Joel Brown*: The prostate adaptive trial (NCT024515621) is instructive here. The original model imagined two categories of sensitive cells that both require testosterone (Zhang et al., 2017). One producing its own, and the other requiring exogenous testosterone. Resistant cells are independent of testosterone and hence unaffected by androgen-focused therapy. In this model, the cost of resistance was assumed to occur primarily through carrying capacity (the maximal cell density) with some contribution of competition coefficients.

The adaptive therapy patients performed better than the contemporaneous controls (median of 33.5 and 14.3 mo radiographic progression free survival, respectively) (Zhang et al., 2021). To be considered for the trial, an initial response to therapy of at least a 50% decline in PSA and putative tumor burden was required. This meant they were responders and hence enjoyed a better prognosis than nonresponders, and nonresponders were not eligible for this trial (Mistry, 2021). The contemporaneous control group was selected from patients receiving continuous therapy and who, like the trial patients, were responders. No nonresponders were included in the control cohort. By way of caveat, this is an important consideration for any trial of adaptive therapy that lacks double-blind, randomized control arms. Most strikingly, some of the adaptive therapy patients performed better than expected from the initial model. At time of writing, four men remain on adaptive therapy after 4–6 y.

Evolutionary theory tells us that adaptive therapy, or more specifically competitive release therapy, works best if the sensitive cells are allowed to grow to the point where both frequency- and density-dependent feedbacks are strong. The frequency-dependent effects offer hope that the sensitive cells are competitively superior to the resistant ones. This cost of resistance can manifest as a reduction in maximum growth rates, reduction in carrying capacity, and the competitive effect of each cell type on the other (Zhang et al., 2022; Strobl et al., 2020; Pressley et al., 2021). A retrospective analysis of the near-finished trial (Zhang et al., 2022), permits parameter estimations, some patient specific, others estimated as patient-wide parameters. The efficacy of adaptive therapy seems best explained by highly asymmetric competition. It was estimated that the sensitive cells (a streamlined model combining the two sensitive cell types into one) have a competitive effect (per cell) on resistant cells that is six times higher than the reverse effect of resistant cells on sensitive ones. As others have noted in their models, such a high asymmetry favoring sensitive cells relative to resistant cells can result in indefinite control and disease containment (Viossat and Noble, 2021).

The prostate adaptive trial does provide some evidence that prolonged sensitivity may outweigh adverse effects of maintaining high tumor burden, but the promise of being able to perpetually control each patient’s disease did not happen. Why not? First, the periodic measurements of PSA on which to base decisions to stop or start therapy meant that patients often dropped more, and sometimes way more than the desired goal of 50%, and vice versa for when therapy was actually restarted. Virtually all models indicate a poorer performance of adaptive therapy with imprecise switch points that overshoot the targeted switch values. The second reason is decisive and it concerns enlisting the needed density-dependence. If the tumor burden and cancer cell populations sizes are well below carrying capacity, then both cell types may enjoy positive fitness in the absence of therapy (Zhang et al., 2017; Strobl et al., 2020; Hansen and Read, 2020a). As the tumor cell populations grow, per capita growth rates decline for both cell types, but with asymmetric competition, the resistant cells will experience negative fitness even as the sensitive cells continue to grow. This is a sweet spot. In this region of high tumor burden, the sensitive cells not only retard but reverse the growth rate of the resistant cells. Our retrospective analysis supports the conclusion of a number of mathematical and theoretical investigations. Namely, adaptive therapy seems to work best if (1) the threshold for ceasing therapy is quite high (80% rather than 50%, for instance), and (2) the overall tumor burden is maintained as high as possible without endangering the patient (Kim et al., 2021; Hansen and Read, 2020b; Viossat and Noble, 2021).

Retrospective analysis of mathematical modeling fit to adaptive patient data (Zhang et al., 2022) suggests that a physician ought to hold off resuming therapy until as late as possible in terms of the recovery of the tumor during a period of no therapy. With retrospective analysis of each patient, we find that failure of indefinite control happened because tumor burdens during periods of no therapy were being kept too low to permit sufficient density dependence to facilitate negative growth rates of the resistant cells. For these patients, therapy was resumed too soon! Yet, there are likely dangers associated with containment strategies aiming for overall high, and persistent tumor burdens. These fall into four important categories: (1) patients becoming symptomatic, (2) the ability of the biomarker to be sufficiently accurate and measurable frequently, (3) subsequent cancer evolution during the period of adaptive therapy, and (4) risk of new metastases.

An adaptive therapy clinical trial on metastatic thyroid cancer (NCT03630120) illustrates two of these concerns. The trial was suspended. The suspension was not mandated by the required stopping criteria, but because of two unanticipated issues. In one of the patients receiving adaptive therapy, there was an initially good response, therapy was stopped, and the tumor burden allowed to recover. However, the patient began to feel pain and other ill effects of the tumor burden before it had recovered to pretreatment level. The patient had become symptomatic at which point either treatment must be resumed or a different course of therapy considered. In another patient, after the resumption of therapy the tumor continued to grow, at least based on the biomarker, with no indication of a future decline (Christine Chung and Joel S. Brown, unpublished data). The small number of patients in both the randomized control arm (continuous therapy) and the adaptive therapy arm precluded meaningful interpretation of the potential efficacy of adaptive therapy. But it pointed to issues of patients becoming symptomatic, of the reliability of the biomarker as an accurate indicator of tumor burden, and of rapid changes in tumor burden and disease disposition that occurred at a faster time-scale than the ability to adjust therapies.

While not yet documented in any clinical trial of adaptive therapy, there remains the concern that high tumor burden, subjected to on and off therapy cycles may incubate additional mutations and adaptations by the cancer cells. For instance, with time, the resistant cancer cells may evolve traits that minimize or even reverse the cost of resistance. A large residual tumor population is more likely than a very small tumor burden to give rise to such mutations that propel the cancer cells to greater levels of malignancy. As of yet, models of adaptive therapy have not considered the risk of additional progressive evolution beyond that expected regarding drug sensitivity.

Current trials and most models of adaptive therapy consider the patient’s total tumor burden even when it is known that the cancer is spread across several or many metastatic sites. As the overall tumor burden shrinks, different lesions may not necessarily respond similarly or proportionately. Furthermore, the fraction of resistant and sensitive cells may differ among lesions, particularly if they are in different tissues. Over the course of adaptive therapy, the tumor burden may become more dispersed among lesions, more concentrated in a lower number of tumors, or, of most concern, metastasize to new sites. In the case of melanoma, whether under standard of care continuous therapy or as an adaptive therapy trial (NCT03543969), there is a risk of the disease metastasizing to the brain even as therapy efficacy may be good elsewhere in the patient’s body. A recent spatial model of the prostate clinical trial imagined dynamics both within and between metastatic sites in response to the on and off cycles of therapy. The model predicts that the adaptive therapy regimen will vary if the disease is represented by many small versus a few large tumors (Gallaher et al., 2022).

As more clinical trials of adaptive therapy emerge from integrating mathematical models with clinical opportunity and need, it will be essential to consider the tradeoffs associated with maintaining relatively large tumor burdens. A large tumor burden may increase the efficiency of an adaptive therapy regimen while increasing the risks of additional progressive evolution, other ill effects of tumor burden, and the appearance of new lesions within the same or different tissues. Balancing these costs and benefits will likely be disease and drug specific, and will require a continued lockstep between mathematical models and empirical studies, data, and observations of patients.

#### Can a mathematical model drive treatment decision-making?

*Mark Robertson-Tessi and Sandy Anderson*: Many models of adaptive therapy are currently hypothesis-generating models with less focus on predictive insight (Enderling and Wolkenhauer, 2020). One of the main goals of personalized therapy is the ability to predict likely tumor dynamics arising from all available treatment options, and then select the most promising. Therefore, there is a great need for clinically suitable predictive mathematical models that track patient-specific tumor dynamics. There are, however, numerous challenges that need to be surmounted for this approach to be broadly successful and able to be scaled to large numbers of patients.

To begin with, clinical data are often sparse and often collected at times of little value to a mathematical model. For example, imaging scans are often collected some time prior to therapy, and then, depending on the cancer, the first follow-up scan occurs after 6–12 wk. Yet, in many models of different diseases, the relevant action that would inform therapeutic decisions via modeling occurs during the immediate aftermath of therapy. Furthermore, the models benefit greatly from multiple data points, so it may take several follow-up scans to narrow the parameterization of a model to determine treatment efficacy and tumor dynamics, by which time the tumor may have progressed and thus no prediction is needed. In a sense, current imaging schedules are designed to detect progression reasonably soon after it occurs, often using the standard RECIST criteria or similar, while balancing the costs of repeated imaging. To move into predictive mathematical modeling, we must rethink how we collect patient data, in that we do not want to simply detect progression, but rather quantify and understand the tumor dynamics prior to progression such that one can inform treatment decisions to avoid it.

A second consideration is the uncertainty of the entire system, from patient to model. Even an optimally designed data collection protocol that would optimize model predictability will still leave significant uncertainties in such predictions. The idea that we will have the data and modeling to precisely predict the future course of a patient’s disease is not realistic, or even possible. Therefore, all predictions must include statistically rigorous uncertainty analyses. The concept of the ‘Phase i’ trial (Kim et al., 2016; Scott, 2012) has been developed as a framework to quantify and apply such uncertainties. The basic principle is to develop cohorts of virtual patients derived from a calibrated mathematical model and apply ‘clinical trials’ to such cohorts with varying regimens of treatment. Much like changes to the standard of care that arise from cohort-to-cohort comparisons, the same approach is used in the virtual cohorts to decide on the treatment that is most likely to succeed. In general, the complete virtual cohort is formed by examining historical data on the disease, both at the individual patient level and via cohort outcomes from clinical trials. Once this global cohort is established from the model, an individual patient can be compared to each virtual patient and matched to those that exhibit similar dynamics as the real patient. This patient-specific virtual subcohort is then subjected to the available therapy options, and their outcomes as a cohort are compared. The results provide decision-support for the treating physician. Using mechanistic models – as opposed to statistical models alone – has the advantage that as more patient data are collected on follow-up visits, the calibration and refinement of the patient’s subcohort can be improved by removing virtual patients that responded differently.

Adaptive therapy strategies are one part of a broader approach to introduce evolutionary principles into dose scheduling to mitigate the evolution of resistance (Gatenby and Brown, 2020b; Noorbakhsh et al., 2020; Belkhir et al., 2021; Stanková et al., 2019). Ongoing or planned evolution-based treatment trials include a trial in rhabdomyosarcoma, which includes both extinction therapy and adaptive therapy arms (NCT04388839) (Reed et al., 2020), adaptive androgen deprivation for castration-sensitive prostate cancer (NCT03511196), adaptive abiraterone or enzalutamide in castration-resistant prostate cancer (ANZadapt; NCT05393791), adaptive administration of BRAF-MEK inhibitors for advanced BRAF mutant melanoma (NCT03543969), adaptive carboplatin in ovarian cancer (ACTOv trial; NCT05080556), adaptive therapy of Vismodegib in advanced basal cell carcinoma (NCT05651828), and a feasibility study (Robertson-Tessi et al., 2023) for implementing evolution-based strategies with the aid of mathematical modeling decision-support (the ‘evolutionary tumor board’ at the Moffitt Cancer Center; NCT04343365). Given the complexity of cancer as an evolutionary disease, many of these trials have been planned with insights gained from mathematical models.

### Concluding remarks

The questions outlined in the sections above can be categorized into the following: integrating the appropriate components into mathematical models (Integrating the appropriate components into mathematical models), the design and validation of dosing protocols (Design and validation of dosing protocols), and challenges and opportunities in clinical translation (Design and validation of dosing protocols). It is our opinion that work addressing these questions should occur concurrently, within an interdisciplinary framework of science.

**Figure 4.**
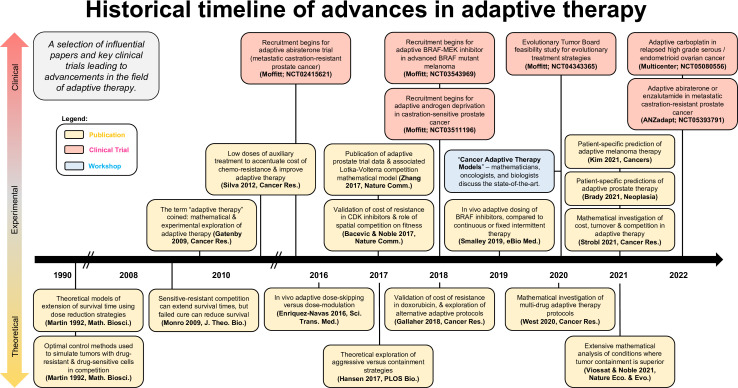
Timeline of advancements in adaptive therapy: a selection of influential papers and key clinical trials leading to advancements in the field of adaptive therapy. This selection includes papers with experimental or clinical adaptive data in addition to well-cited theoretical publications.

Figure 4 shows a selection of influential papers and key clinical trials leading to advancements in the field of adaptive or evolution-based treatment. This figure illustrates that experimental and clinical investigation of adaptive therapy has progressed synergistically with mathematical and computational modeling. Perhaps the most important challenge concerns the communication between different disciplines: the practice of oncology, the theories of ecology and evolution, and the application of mathematical models to data were not historically in sync in terms of possibilities for both practice and outcome. An integrated team science approach focused on gaining a deeper understanding of each disease and the implications of each decision during treatment is key for the future success of an evolution-based cancer management.
